# Clinical characteristics of 134 convalescent patients with COVID-19 in Guizhou, China

**DOI:** 10.1186/s12931-020-01580-0

**Published:** 2020-11-26

**Authors:** Siqin Zhang, Lin Liu, Bin Yang, Rou Li, Jianhua Luo, Jing Huang, Yanjun Long, Ying Huang, Jianping Zhou, Yan Zha, Xiangyan Zhang

**Affiliations:** 1 Department of Endocrinology and Metabolism, Guizhou Provincial People’s Hospital, Guiyang, 550002 Guizhou China; 2 Department of Respiratory and Critical Medicine, Guizhou Provincial People’s Hospital, No. 83, Zhongshan East Road, Guiyang, 550002 Guizhou China; 3 Department of Central Laboratory, Guizhou Provincial People’s Hospital, Guiyang, 550002 Guizhou China; 4 School of Medicine, Guizhou University, Guiyang, 550000 Guizhou China; 5 Department of Cardiology, Guizhou Provincial People’s Hospital, Guiyang, 550002 Guizhou China; 6 Department of Nephrology, Institute of Nephritic and Urinary Disease, Guizhou Provincial People’s Hospital, No. 83, Zhongshan East Road, Guiyang, 550002 Guizhou China; 7 Department of Psychology, Guizhou Provincial People’s Hospital, Guiyang, 550002 Guizhou China; 8 Department of Pediatrics, Guizhou Provincial People’s Hospital, Guiyang, 550002 Guizhou China

**Keywords:** Coronavirus disease 2019 (COVID-19), Clinical features, Disease severity, Pneumonia

## Abstract

**Background:**

Previous studies have focused on the clinical characteristics of hospitalized patients with the novel 2019 coronavirus disease (COVID-19). Limited data are available for convalescent patients. This study aimed to evaluate the clinical characteristics of discharged COVID-19 patients.

**Methods:**

In this retrospective study, we extracted data for 134 convalescent patients with COVID-19 in Guizhou Provincial Staff Hospital from February 15 to March 31, 2020. Cases were analyzed on the basis of demographic, clinical, and laboratory data as well as radiological features.

**Results:**

Of 134 convalescent patients with COVID-19, 19 (14.2%) were severe cases, while 115 (85.8%) were non-severe cases. The median patient age was 33 years (IQR, 21.8 to 46.3), and the cohort included 69 men and 65 women. Compared with non-severe cases, severe patients were older and had more chronic comorbidities, especially hypertension, diabetes, and thyroid disease (P < 0.05). Leukopenia was present in 32.1% of the convalescent patients and lymphocytopenia was present in 6.7%, both of which were more common in severe patients. 48 (35.8%) of discharged patients had elevated levels of alanine aminotransferase, which was more common in adults than in children (40.2% vs 13.6%, P = 0.018). A normal chest CT was found in 61 (45.5%) patients during rehabilitation. Severe patients had more ground-glass opacity, bilateral patchy shadowing, and fibrosis. No significant differences were observed in the positive rate of IgG and/or IgM antibodies between severe and non-severe patients.

**Conclusion:**

Leukopenia, lymphopenia, ground-glass opacity, and fibrosis are common in discharged severe COVID-19 patients, and liver injury is common in discharged adult patients. We suggest physicians develop follow-up treatment plans based on the different clinical characteristics of convalescent patients.

## Background

Since December 2019, pneumonia cases of unknown cause have been reported [1]. A novel coronavirus specimen isolated from the lower respiratory tract was identified as the causative pathogen, and officially named Severe Acute Respiratory Syndrome Coronavirus-2 (SARS-CoV-2) [2]. This virus belongs to the β genus of coronaviruses, and shares 88% homology with bat-derived severe acute respiratory syndrome (SARS)-like coronaviruses [3]. Studies indicate that bats may be the natural host of this coronavirus. The 2019 coronavirus disease (COVID-19) is a newly recognized illness that has spread rapidly in China and internationally [4–6]. As of July 5, 2020, more than 11 million laboratory-confirmed cases and 528,204 deaths from COVID-19 had been documented globally, according to the official website of the World Health Organization. Thus, COVID-19 infection poses a serious health threat worldwide.

Previous studies have described the clinical characteristics of hospitalized patients with COVID-19 [7, 8]. Guan et al. [9] reported the clinical findings for 1099 confirmed patients in China. The main clinical features included fever, cough, lymphocytopenia, and ground-glass opacity in the lungs observed in radiologic imaging. Shock, acute respiratory distress syndrome (ARDS), organ failure, and death occurred in severe cases [10]. Compared with non-severe patients, severe cases occurred in patients who were older in age, had a higher prevalence of hypertension, and had more abnormal indicators in laboratory tests [11]. With increasing numbers of discharged COVID-19 patients, medical care and follow-up observation after discharge are necessary. Nevertheless, few studies have been conducted to describe the clinical characteristics in convalescent patients with COVID-19.

This study aimed to analyze the clinical characteristics, laboratory data, and radiologic findings of discharged patients with COVID-19, and compare the differences between severe and non-severe groups. The differences in clinical features in children and adults were also compared. Our finding might be helpful for clinicians who follow-up treat and manage discharged COVID-19 patients.

## Methods

### Study participants and design

We conducted a retrospective study of 134 convalescent patients with COVID-19. All patients met the following discharge criteria according to the Novel Coronavirus Infection Pneumonia Diagnosis and Treatment Standards (7th edition) [12]: (1) normal temperature for more than 3 days; (2) respiratory symptoms improved significantly; (3) substantial improvement of acute exudative lesions on chest radiography; (4) two consecutively negative results of RT-PCR tests with at least a one day interval between tests. Taking into account the risk of reinfection, all discharged patients were further isolated and underwent medical observation in Guizhou Provincial Staff Hospital between February 15 and March 31, 2020. Demographic data, clinical variables, laboratory parameters, and chest CT scans were collected during rehabilitation for further analysis. This study was approved by the Ethics Committee of Guizhou Provincial People's Hospital, and written informed consent was waived.

### Data collection

We reviewed clinical records, laboratory results, and chest CT findings for all patients recovered from COVID-19. The laboratory tests and CT scans were performed 2 weeks after discharge. Improvement of laboratory results were assessed at a median follow-up time of 4 weeks after discharge. Standardized data collection forms were used to record patient age, gender, epidemiological data, comorbidities, laboratory results, and radiological features. Two investigators independently filled and checked the data forms to ensure data accuracy. Exposure history within 14 days before onset of the disease was classified as a history of residence in Hubei, Hubei tourism for non-Hubei residents, contact with people from Hubei, and contact with other confirmed patients.

The severity of COVID-19 was defined according to the Novel Coronavirus Infection Pneumonia Diagnosis and Treatment Standards (7th edition) [12]. Asymptomatic cases were defined as individuals infected by SARS-CoV-2 with no clinical manifestations. Mild cases were defined as confirmed cases with mild clinical symptoms and no radiographic findings of pneumonia. Common cases were defined as patients infected by SARS-CoV-2 with fever, respiratory symptoms, and radiographic findings of pneumonia. Severe cases were defined as having one of the following criteria: (1) respiratory distress with respiratory frequency ≥ 30 times/min; (2) oxygen saturation ≤ 93% at rest; (3) arterial partial oxygen pressure (PaO2)/oxygen absorption concentration (FiO2) ≤ 300 mmHg. Critical cases were defined as having one of the following criteria: (1) respiratory failure and the need for mechanical ventilation; (2) shock; (3) other organ failure and requirement for ICU treatment. All cases were divided into severe and non-severe groups for comparison. The severe group included severe and critical cases, and the non-severe group consisted of asymptomatic, mild, and common cases. Furthermore, the clinical characteristics and laboratory findings between adults and children were also compared. Patients less than 18 years were classified as children, while patients ≥ 18 years were classified as adults.

Laboratory tests conducted for convalescent patients included analysis of levels of white blood cells (WBC), lymphocytes, neutrophils, liver function, creatinine, lactate dehydrogenase (LDH), C-reactive protein (CRP), erythrocyte sedimentation rate (ESR), procalcitonin, creatine kinase isoenzyme (CKMB), and D-dimer. Antibodies against SARS-CoV-2 in the serum were also analyzed, including immunoglobulin G (IgG) and immunoglobulin M (IgM). The results were expressed as positive or negative. All patients underwent chest imaging examination during rehabilitation, and imaging findings were evaluated by experienced radiologists. The imaging features included ground-glass opacity, local patchy shadowing, bilateral patchy shadowing, interstitial abnormalities, and fibrosis.

### Statistical analysis

Continuous variables (non-normal distribution) were described as median and interquartile range (IQR) values, and categorical variables were described as frequency counts and percentages. Analyses of continuous variables between two groups were performed using the Mann–Whitney test. Proportions for categorical data were assessed using a chi-square test. When the data were limited, Fisher’s exact test was performed. Statistical analysis was conducted with the Statistical Product and Service Solution (SPSS) 22.0 (IBM, USA). P values < 0.05 were considered to indicate statistical significance.

## Results

### Demographic and clinical characteristics

A total of 134 convalescent patients with COVID-19 were enrolled in this study. All enrolled patients met discharge criteria and were admitted to Guizhou Provincial Staff Hospital for isolated medical observation. The demographic and clinical characteristics of the patients are shown in Table 1. The median age of the patients was 33 years (IQR, 21.8 to 46.3), and 69 (51.5%) were men. 41 (30.6%) patients were residents of Hubei, while 32 (23.9%) patients lived outside Hubei and had visited the province within past 14 days. A history of contact with people from Hubei was documented in 20.1%, while 34 (25.4%) patients had no exposure history linked to Hubei. 72 patients (53.7%) were cluster cases. Of the 134 patients, 25 (18.7%) had at least one comorbidity. Hypertension [14 (10.4%)] and diabetes [10 (7.5%)] were the most common comorbidities. All patients had no fever, cough, sputum production, or fatigue during the rehabilitation period, and displayed no abnormal signs in the lungs or throat.

**Table 1:** Clinical characteristics of COVID-19 patients according to disease severity

	Total N = 134	Severe N = 19	Non-severe N = 115	P value
Age, median (IQR), y	33.0 (21.8–46.3)	53.0 (37.0–61.0)	30.0 (20.0–42.0)	** < 0.001**
Sex, male n (%)	69 (51.5%)	11 (57.9%)	58 (50.4%)	0.547
*Exposure history within past 14 days, n (%)*				
Living in Hubei	41 (30.6%)	8 (42.1%)	33 (28.7%)	**0.007**
Recently visited Hubei	32 (23.9%)	4 (21.1%)	28 (24.3%)	
Contact with people from Hubei	27 (20.1%)	7 (36.8%)	20 (17.4%)	
Contact with other confirmed patients	34 (25.4%)	0	34 (29.6%)	
*Cluster cases, n (%)*	72 (53.7%)	11 (57.9%)	61 (53.0%)	0.694
*Comorbidities, n (%)*				
Any	25 (18.7%)	12 (63.2%)	13 (11.3%)	** < 0.001**
Diabetes	10 (7.5%)	6 (31.6%)	4 (3.5%)	** < 0.001**
Hypertension	14 (10.4%)	7 (36.8%)	7 (6.1%)	** < 0.001**
Coronary heart disease	1 (0.7%)	1 (5.3%)	0	0.142
Chronic bronchitis	1 (0.7%)	1 (5.3%)	0	0.142
Gout	2 (1.5%)	1 (5.3%)	1 (0.9%)	0.264
Cancer	1 (0.7%)	0	1 (0.9%)	> 0.99
AIDS	1 (0.7%)	0	1 (0.9%)	> 0.99
Thyroid disease	2 (1.5%)	2 (10.5%)	0	**0.019**

Bold values indicate that P value is less than 0.05

*COVID-19* coronavirus disease 2019, *IQR* interquartile range, *AIDS* acquired immune deficiency syndrome

In terms of the degree of severity, 19 (14.2%) patients were severe cases, and 115 (85.8%) were non-severe cases. Patients with severe disease were significantly older than those with non-severe disease (53 years vs. 30 years, P < 0.001), and were more likely to have exposure history linked to Hubei. Compared to the non-severe group, severe patients had more chronic comorbidities, especially hypertension, diabetes, and thyroid disease (all P < 0.05).

The clinical features of children and adults with COVID-19 are shown in Table 2. A total of 134 convalescent patients included 112 adult cases and 22 child cases. The median age of the pediatric group was 13 years (IQR, 3.8 to 12.5), while the median age of the adult cases was 56 years (IQR, 25.3 to 49.0). There were no significant differences in gender distribution or exposure history between the two groups. All pediatric cases were those not categorized as severe. 19 patients in the adult group were severe COVID-19 cases, making severe cases much more common in the adult group compared to children (17% vs. 0%, P = 0.037). Compared to the adult group, children had more cluster cases (81.8% vs. 48.2%, P = 0.004).

**Table 2:** Clinical characteristics of children and adults with COVID-19

	Total N = 134	Children N = 22	Adults N = 112	P value
Age, median (IQR), y	33.0 (21.8–46.3)	9.5 (3.8–12.5)	37.0 (25.3–49.0)	** < 0.001**
Sex, male n (%)	69 (51.5%)	13 (59.1%)	56 (50%)	0.435
*Exposure history within past 14 days, n (%)*				
Living in Hubei	41 (30.6%)	4 (18.2%)	37 (33.0%)	0.372
Recently visited Hubei	32 (23.9%)	8 (36.4%)	24 (21.4%)	
Contact with people from Hubei	27 (20.1%)	4 (18.2%)	23 (20.5%)	
Contact with other confirmed patients	34 (25.4%)	6 (27.3%)	28 (25.0%)	
*Cluster cases, n (%)*	*72 (53.7%)*	*18 (81.8%)*	*54 (48.2%)*	*0.004*
*Clinical classifications, n (%)*				
Non-severe	115 (85.8%)	22 (100%)	93 (83.0%)	**0.037**
Severe	19 (14.2%)	0	19 (17%)	

Bold values indicate that P value is less than 0.05

*COVID-19* coronavirus disease 2019, *IQR* interquartile range

### Laboratory and radiologic findings

**Fig. 1:**
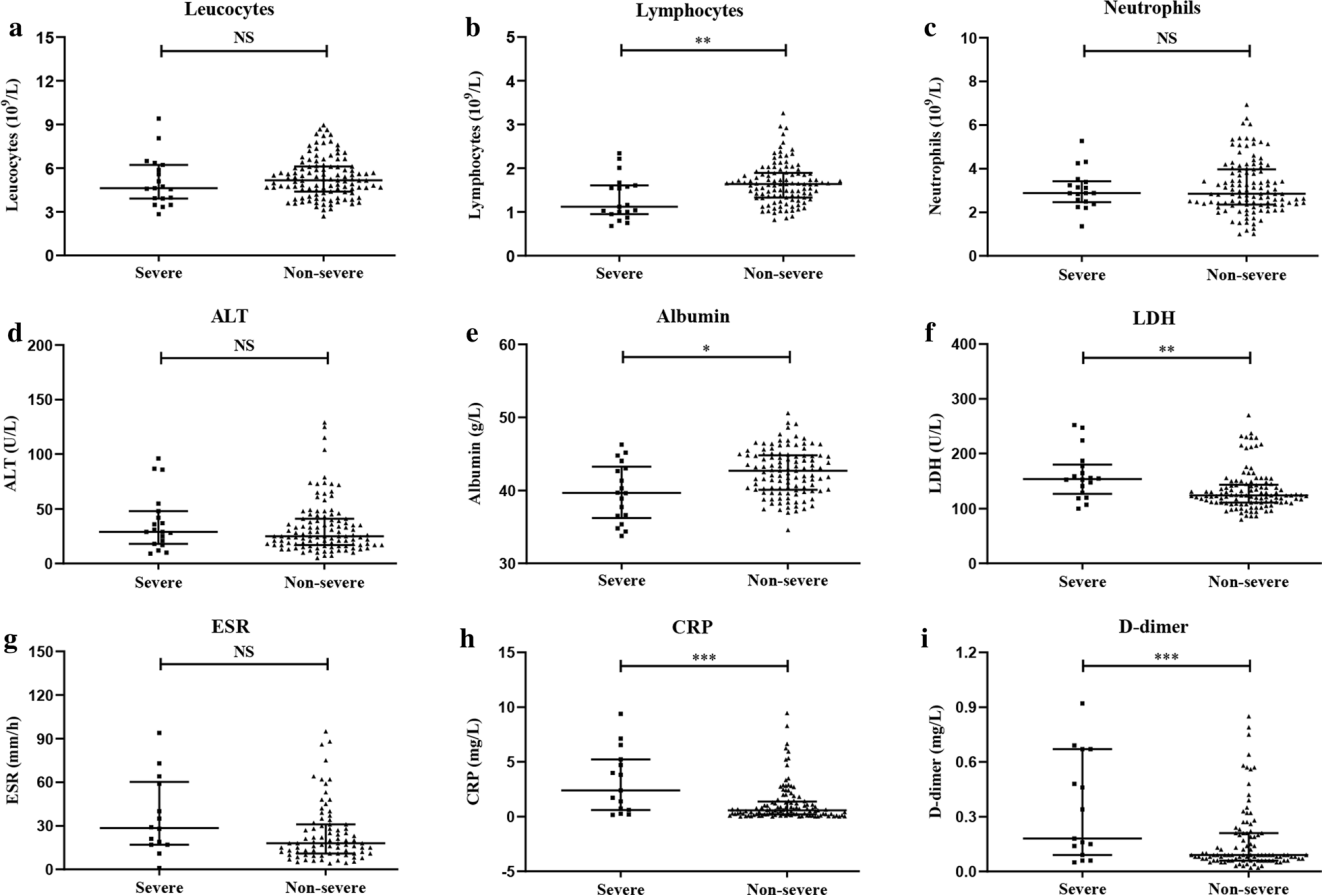
Laboratory results of patients with COVID-19 during convalescence. Patient levels of **a** leukocytes, **b** lymphocytes, **c** neutrophils, **d** alanine aminotransferase (ALT), **e** albumin; **f** lactate dehydrogenase (LDH), **g** erythrocyte sedimentation rate (ESR), **h** C-reactive protein (CRP), and **i** D-dimer. *P < 0.05, **P < 0.01 and ***P < 0.001; *NS* not significant

The laboratory and radiologic findings of severe and non-severe patients two weeks after discharge are shown in Table 3 and Fig. 1. Overall, leukopenia was present in 32.1% of convalescent patients, lymphocytopenia was present in 6.7%, and neutropenia was present in 24.6%. 48 (35.8%) patients had elevated levels of alanine aminotransferase (ALT). Common infection-related indicators of abnormalities included increased ESR (64.9%) and CRP (23.9%). Less common indicators were elevated levels of total bilirubin, creatinine, CKMB, LDH, troponin, and procalcitonin. Compared with non-severe cases, patients with severe disease had lower levels of lymphocytes and albumin, as well as higher levels of ESR, CRP, D-dimer, and LDH (P < 0.05). The laboratory test results of children and adults were also analyzed (Table 4). The results of this analysis showed that pediatric patients in the rehabilitation period were less likely to have elevated ALT, ESR, and CKMB when compared with adults.

**Table 3:** Laboratory and radiologic findings of severe and non-severe patients with COVID-19 during convalescence

	Total N = 134	Severe N = 19	Non-severe N = 115	P value
*Blood routine*				
Leukopenia	43/134 (32.1%)	11/19 (57.9%)	32/115 (27.8%)	**0.009**
Lymphopenia	9/134 (6.7%)	5/19 (26.3%)	4/115 (3.5%)	**0.001**
Neutropenia	33/134 (24.6%)	6/19 (31.6%)	27/115 (23.5%)	0.637
*Blood biochemistry*				
ALT increased	48/134 (35.8%)	7/19 (36.8%)	41/115 (35.7%)	0.920
AST increased	19/134 (14.2%)	2/19 (10.5%)	17/115 (14.8%)	0.890
Total bilirubin increased	3/134 (2.2%)	0/19	3/115 (2.6%)	> 0.99
Albumin decreased	7/134 (5.2%)	4/19 (21.1%)	3/115 (2.6%)	**0.005**
Creatinine increased	1/134 (0.7%)	0/19	1/115 (0.9%)	> 0.99
CKMB increased	15/134 (11.2%)	1/19 (5.3%)	14/115 (12.2%)	0.622
LDH increased	4/134 (3.0%)	3/19 (15.8%)	1/115 (0.9%)	**0.009**
Troponin increased	0/134	0/19	0/115	
*Inflammation-related indicators*				
ESR increased	63/97 (64.9%)	15/16 (93.8%)	48/81 (59.3%)	**0.008**
CRP increased	32/134 (23.9%)	9/19 (47.4%)	23/115 (20.0%)	**0.021**
PCT increased	3/127 (2.4%)	0/19	3/108 (2.8%)	> 0.99
*Coagulation function*				
D-dimer increased	21/120 (17.5%)	7/18 (38.9%)	14/102 (13.7%)	**0.024**
*Chest CT findings*				
Normal	61 (45.5%)	2 (10.5%)	59 (51.3%)	**0.001**
Ground-glass opacity	40 (29.9%)	11 (57.9%)	29 (25.2%)	**0.004**
Local patchy shadowing	13 (9.7%)	1 (5.3%)	12 (10.4%)	0.774
Bilateral patchy shadowing	25 (18.7%)	11 (57.9%)	14 (12.2%)	> 0.99
Interstitial abnormalities	4 (3.0%)	1 (5.3%)	3 (2.6%)	0.462
Fibrosis	36 (26.9%)	11 (57.9%)	25 (21.7%)	**0.001**

Bold values indicate that P value is less than 0.05

*COVID-19* coronavirus disease 2019, *ALT* alanine aminotransferase, *AST* aspartate aminotransferase, *CKMB* creatine kinase isoenzyme, *LDH* lactate dehydrogenase, *ESR* erythrocyte sedimentation rate, *CRP* C-reactive protein, *PCT* procalcitonin

**Table 4:** Laboratory and radiologic findings of children and adults with COVID-19 during convalescence

	Total N = 134	Children N = 22	Adults N = 112	P value
*Blood routine*				
Leukopenia	43/134 (32.1%)	6/22 (27.3%)	37/112 (33.0%)	0.597
Lymphopenia	9/134 (6.7%)	0/22	9/112 (8.0%)	0.362
Neutropenia	33/134 (24.6%)	9/22 (40.9%)	24/112 (21.4%)	0.053
*Blood biochemistry*				
ALT increased	48/134 (35.8%)	3/22 (13.6%)	45/112 (40.2%)	**0.018**
AST increased	19/134 (14.2%)	3/22 (13.6%)	16/112 (14.3%)	> 0.99
Total bilirubin increased	3/134 (2.2%)	0/22	3/112 (2.7%)	> 0.99
Albumin decreased	7/134 (5.2%)	1/22 (4.5%)	6/112 (5.4%)	> 0.99
Creatinine increased	1/134 (0.7%)	0/22	1/112 (0.9%)	> 0.99
CKMB increased	15/134 (11.2%)	6/22 (27.3%)	9/112 (8.0%)	**0.025**
LDH increased	4/134 (3.0%)	0/22	4/112 (3.6%)	> 0.99
Troponin increased	0/130	0/21	0/109	
*Inflammation-related indicators*				
ESR increased	63/97 (64.9%)	6/19 (31.6%)	57/78 (73.1%)	**0.001**
CRP increased	32/134 (23.9%)	3/22 (13.6%)	29/112 (25.9%)	0.218
PCT increased	3/126 (2.4%)	1/21 (4.8%)	2/105 (1.9%)	0.424
*Coagulation function*				
D-dimer increased	21/120 (17.5%)	3/22 (13.6%)	18/98 (18.4%)	0.828
*Chest CT findings*				
Normal	61 (45.5%)	16 (72.7%)	45 (40.2%)	**0.005**
Ground-glass opacity	40 (29.9%)	1 (4.5%)	39 (34.8%)	**0.005**
Local patchy shadowing	13 (9.7%)	2 (9.1%)	11 (9.8%)	> 0.99
Bilateral patchy shadowing	25 (18.7%)	0	25 (22.3%)	**0.014**
Interstitial abnormalities	4 (3.0%)	2 (9.1%)	2 (1.8%)	0.126
Fibrosis	36 (26.9%)	1 (4.5%)	35 (31.3%)	**0.010**

Bold values indicate that P value is less than 0.05

*COVID-19* coronavirus disease 2019, *ALT* alanine aminotransferase, *AST* aspartate aminotransferase, *CKMB* creatine kinase isoenzyme, *LDH* lactate dehydrogenase, *ESR* erythrocyte sedimentation rate, *CRP* C-reactive protein, *PCT* procalcitonin

The abnormalities in laboratory tests were gradually resolved in the four weeks follow-up after discharge. The number of leukopenia reduced from 43 (32.1%) to 9 (6.7%). The patients of lymphocytopenia changed from 9 (6.7%) to 1 (0.7%), and neutropenia decreased from 33 (24.6%) to 12 (9.0%). Among 48 patients with elevated ALT, 37 (77.1%) patients returned to normal liver function. Also, 38.1% of patients with elevated ESR and 46.9% of patients with increased CRP turned to normal four weeks after discharge.

Of the 134 CT scans that were performed during rehabilitation, 54.5% revealed abnormal results, which were significantly improved when compared with those observed at the onset of infection. The most common abnormalities observed on chest CT scans were ground-glass opacity (29.9%) and fibrosis (26.9%), while interstitial abnormalities were rare (3.0%). Patients with severe disease had more ground-glass opacity, bilateral patchy shadowing, and fibrosis than non-severe patients (P < 0.05). The prevalence of local patchy shadowing and interstitial abnormality between severe and non-severe cases was similar. Compared with adult patients, children had significantly less radiologic abnormalities, especially in terms of ground-glass opacity (4.5% vs. 34.8%, P = 0.005), bilateral patchy shadowing (0% vs. 22.3%, P = 0.014), and fibrosis (4.5% vs. 31.3%, P = 0.010).

### Antibody responses to SARS-CoV-2 in convalescent patients

Of the 134 convalescent patients, 55 were tested for antibodies against SARS-CoV-2. The patients were classified into two groups: non-severe (49 cases, 89.1%) and severe (6 cases, 10.9%). Overall, IgG was detected in 38 cases (69.1%), IgM was detected in 3 cases (5.5%), and both IgG and IgM were detected in 5.5% of cases. In severe patients, 5 out of 6 patients generated virus-specific IgG antibodies, and none of the patients displayed an IgM antibody response. Among the 49 non-severe patients, 30 patients were IgG (+) and IgM (−), and 3 patients were positive for IgG and IgM. There was no significant difference in the positive rate of IgG and/or IgM antibodies between severe and non-severe patients (Table 5). A total of 55 recovered patients included 7 children and 48 adults. IgG antibody responses were detected in 5 pediatric cases (71.4%) and 33 adult cases (68.8%). The positive rate of IgG and/or IgM antibodies in children and adults was similar (P > 0.05, Table 5).

**Table 5:** Antibody responses to SARS-CoV-2 in convalescent patients

	Total N = 55	Disease severity		P value	Age		P value
		Severe N = 6	Non-severe N = 49		Children N = 7	Adults N = 48	
IgM (-),IgG (+)	35 (63.6%)	5 (83.3%)	30 (61.2%)	0.399	5 (71.4%)	30 (62.5%)	> 0.99
IgM ( +),IgG (+)	3 (5.5%)	0	3 (6.1%)	> 0.99	0	3 (6.3%)	> 0.99
IgM (-),IgG (-)	17 (30.9%)	1 (16.7%)	16 (32.7%)	0.654	2 (28.6%)	15 (31.3%)	> 0.99

*SARS-CoV-2* Severe Acute Respiratory Syndrome Coronavirus-2, *IgM* immunoglobulin M, *IgG* immunoglobulin G

## Discussion

This is a retrospective study on the clinical characteristics of COVID-19 during rehabilitation, including data from 134 recovered patients. 19 patients were severe cases and 115 were non-severe cases. The patients with severe disease were older and had more underlying comorbidities than those with non-severe disease. Lymphopenia was more common in discharged severe patients. Adult patients were more prone to liver dysfunction than children. In addition, half of the convalescent patients had abnormal radiologic images, most commonly presenting with ground-glass opacity and fibrosis.

We observed that in this cohort, severe patients with COVID-19 were older than non-severe cases. Consistent with our study, Wang et al. [13] found patients who required ICU treatment were older than those who did not. Another study also observed significantly increased average age in severely affected patients [14]. A possible explanation for this might be low immune function in elderly patients that contributes to poor outcomes. Therefore, elderly patients require more attention and protection. In addition, all pediatric patients in this study were non-severe cases. Similar to our findings, previous studies showed that the clinical symptoms and disease severity of children with COVD-19 were milder compared with adults [15, 16]. It is known that angiotensin converting enzyme 2 (ACE2) is the receptor that allows SARS-CoV-2 to enter host cells [17]. The number of ACE2 receptors present on host cells determines susceptibility to COVID-19 to a certain extent. Compared with adults, the expression of ACE2 in the nasal epithelium was determined to be lower in younger children [18]. This may be responsible, at least in part, for the less severe condition of children with COVID-19.

Almost all severe patients had an exposure history linked to Hubei, including recent tourism, history of residency, or contact with people from Hubei. 29.6% of non-severe patients had no exposure history to Hubei and had contact with other confirmed patients. These findings echo recent reports that found the proportion of severe cases was higher than that of non-severe cases for patients living in Wuhan [9]. This suggests that patients with an exposure history linked to Hubei may be more likely to develop severe illness. A decrease in virus virulence during intergenerational transmission is a possible explanation for this phenomenon [11]. In this study, children had a significantly higher proportion of cluster cases than adults. This result is consistent with previous findings, which found that all pediatric cases were related to family clusters [16]. A likely reason for this finding is minimal social activity among children due in part to school closures during the outbreak.

Our study found that 25 (18.7%) patients had at least one comorbidity, and the proportion of severe patients with comorbidities was higher than that among the non-severe group. Hypertension and diabetes remained the most common comorbidities observed. A multicenter research study found that hypertension and diabetes were independent risk factors for poor patient outcome after adjusting for age and smoking status, and the greater the number of comorbidities, the worse the COVID-19 prognosis [19]. Consistent with our finding, a study on another beta genus coronavirus, Middle East Respiratory syndrome (MERS), indicated that diabetes was significantly associated with poor prognosis [20]. The underlying mechanisms for diabetes leading to poor prognosis of MERS infection were dysregulated immune response and prolonged lung inflammation, which were identified using diabetic mice models [21]. Therefore, we speculate that similarly to MERS, immune dysfunction and prolonged inflammation may be possible underlying causes of poor outcomes in COVID-19 patients with comorbidities. Further research is needed to confirm the specific mechanisms, and clinicians should pay more attention to the treatment and protection of COVID-19 patients with comorbidities.

In terms of laboratory tests during rehabilitation, 43 (32.1%) patients were found to have a low absolute value of leukocytes. Leukopenia, lymphopenia, and elevated ESR and CRP were more common in recovered patients with severe COVID-19 than those who had non-severe cases. Similar to our finding, previous studies have shown that lymphopenia and elevated CRP are indicators of poor outcome for COVID-19 patients [22, 23]. The immune system is triggered by viral infections. Lymphocytes, especially T lymphocytes, play a vital role in regulating the immune response to SARS-CoV-2 infection, and this can cause changes in the levels of peripheral blood leukocytes and lymphocytes [24]. Furthermore, compared with the non-severe group, we observed lower levels of albumin and higher levels of LDH and D-dimer in the severe group. Consistent with our study, Shen et al. [22] observed increased D-dimer and LDH levels were correlated with a worse prognosis in COVD-19 patients. High levels of D-dimer indicate a hypercoagulable state, which may cause pulmonary thrombosis [7]. Albumin is an indicator that reflects nutritional status, and decreased albumin levels indicates the body has lower levels of resistance to viral infection [25]. We suggest that patients need more nutritional support to enhance resistance during rehabilitation.

In our study, 48 (35.8%) discharged patients had elevated ALT levels. The proportion of liver injury was significantly lower in children than adults, but there was no difference observed between severe and non-severe patients. Inconsistent with our results, a recent meta-analysis showed severe patients had higher levels of ALT [26]. The reason for this difference might be that the laboratory tests in the meta-analysis were conducted during hospitalization, while our laboratory results were conducted during the rehabilitation period, and patient liver function might be restored after treatment. The lower risk of liver dysfunction in children during rehabilitation may be related to mild illness and increased repair capability. The pathological features of liver biopsy in patients with COVID-19 were microvesicular steatosis and mild lobular and portal activity, suggesting that liver injury may be related to antiviral drug or SARS-CoV-2 infection [27]. The underlying mechanism of liver injury caused by SARS-CoV-2 infection may be that the virus binds to the ACE2 receptors of bile duct cells [28]. Based on this result, we suggest that adult patients should be dynamically monitored for liver function and treated accordingly during the rehabilitation period.

In the recovery period, a normal chest CT result was observed in 61 (45.5%) patients with COVID-19. This was significantly higher than the 17.9% normal CT in non-severe cases and 2.9% in severe cases on admission reported in a previous study [9]. Consistent with chest CT findings during hospitalization [9], in this study ground-glass opacity was the most common imaging abnormality observed during rehabilitation. In addition, we found that 26.9% of discharged patients had pulmonary fibrosis, and the proportion of fibrosis in severe patients was higher than that of non-severe cases. ACE2, the receptor of SARS-CoV-2, is mainly expressed by type II alveolar epithelial cells. SARS-CoV-2 infection causes damage to alveolar epithelial cells and excessively activates transforming growth factor-β (TGF- β)-related pathways, which contributes to pulmonary fibrosis [29]. Therefore, clinicians should pay attention to the lung function status of severe patients with COVID-19 following discharge. Further studies on how to reduce the incidence of pulmonary fibrosis and improving lung function in recovered patients are needed.

Similar to a recent study [30], we found that the antibody positivity rate was not significantly different between severe and non-severe patients with COVID-19. Our data showed that positive rates of IgG and IgM were 69.1% and 5.5%, respectively. Inconsistent with our study, Zhao et al. [31] found the seroconversion rate for IgM was 82.7%, which was higher than our results. The seroconversion rate for IgG was similar across the studies. A possible explanation for this might be that the antibody tests were conducted at different stages of the disease. IgM against SARS-CoV-2 is produced around 1 week after symptom onset and peaks in 2–3 weeks, and then gradually decreases [30]. Thus, recovered patients had lower positive rates of IgM in this study. IgG is produced later than IgM and is typically maintained at a high level for 2 months [30]. Therefore, the levels of IgG and IgM can roughly reflect the disease stage.

This study has several limitations. First, only 55 of the 134 convalescent patients were tested for antibodies, and the antibodies were only analyzed in terms of positivity rate and not at a more detailed level, so factors that influence antibody levels or dynamic evolution of antibodies could not be analyzed. Second, the sample size is limited in our study, especially for severe patients. Therefore, differences in clinical characteristics between severe and non-severe patients may be overlooked.

## Conclusion

In summary, patients with old age and underlying comorbidities are more likely to have severe illness. Leukopenia, lymphopenia, ground-glass opacity, and fibrosis are common in discharged severe patients, and liver injury is common in discharged adult patients. We suggest formulating a follow-up treatment plan for different severity COVID-19 populations after discharge based on clinical characteristics observed during rehabilitation and recovery from infection.

## Data Availability

All data generated or analysed during this study are included in this published article.

## Abbreviations

COVID-19: 2019 Coronavirus disease
SARS-CoV-2: Severe acute respiratory syndrome Coronavirus-2
SARS: Severe acute respiratory syndrome
MERS: Middle East respiratory syndrome
ARDS: Acute respiratory distress syndrome
PaO2: Arterial partial oxygen pressure
FiO2: Oxygen absorption concentration
WBC: White blood cells
ALT: Alanine aminotransferase
LDH: Lactate dehydrogenase
CRP: C-reactive protein
ESR: Erythrocyte sedimentation rate
CKMB: Creatine kinase isoenzyme
IgG: Immunoglobulin G
IgM: Immunoglobulin M
ACE2: Angiotensin converting enzyme 2
TGF- β: Transforming growth factor-β
IQR: Interquartile range
SPSS: Statistical product and service solution
